# Retraction: Efficacy of fertilizing method for different potash sources in cotton (*Gossypium hirsutum* L.) nutrition under arid climatic conditions

**DOI:** 10.1371/journal.pone.0277637

**Published:** 2022-11-16

**Authors:** The PLOS ONE Editors

The *PLOS ONE* Editors retract this article [1] because it was identified as one of a series of submissions for which we have concerns about authorship, competing interests, and peer review. We regret that the issues were not addressed prior to the article’s publication.

AN, SUA, and AW did not agree with the retraction. MH, AFT, MN, and AS either did not respond directly or could not be reached.
